# Urchin-like WO_3_ Particles Form Honeycomb-like Structured PLA/WO_3_ Nanocomposites with Enhanced Crystallinity, Thermal Stability, Rheological, and UV-Blocking and Antifungal Activity

**DOI:** 10.3390/polym16192702

**Published:** 2024-09-24

**Authors:** Sihem Daikhi, Salim Hammani, Soumia Guerziz, Huda Alsaeedi, Syreina Sayegh, Mikhael Bechlany, Ahmed Barhoum

**Affiliations:** 1 Laboratoire de Chimie Physique Moléculaire et Macromoléculaire, Faculté de Science, Université de Blida 1, Blida 09000, Algeria; s_daikhi@yahoo.com (S.D.); soumia.guerziz@gmail.com (S.G.); 2 Department of Chemistry, College of Science, King Saud University, Riyadh 11451, Saudi Arabia; halsaeedi@ksu.edu.sa; 3 Institut Européen des Membranes, IEM, UMR-5635, University Montpellier, ENSCM, CNRS, Place Eugene Bataillon, F-34095 Montpellier, France; syreina.sayegh@gmail.com (S.S.); mikhael.bechelany@umontpellier.fr (M.B.); 4 Functional Materials Group, Gulf University for Science and Technology (GUST), Masjid Al Aqsa Street, Mubarak Al-Abdullah 32093, Kuwait; 5 NanoStruc Research Group, Chemistry Department, Faculty of Science, Helwan University, Cairo 4034572, Egypt

**Keywords:** PLA/WO_3_ nanocomposites, honeycomb-like morphology, structural enhancement, thermal stability, optical properties, rheological behavior, crystallinity improvement, antifungal activity

## Abstract

The development of poly(lactic acid) (PLA) nanocomposites incorporating urchin-like WO_3_ particles through a cost-effective solution-casting method has led to significant enhancements in structural, thermal, optical, and rheological properties. The incorporation of these WO_3_ particles up to 7 wt% resulted in the formation of an irregular honeycomb-like morphology with broad pore sizes ranging from 14.1 to 24.7 µm, as confirmed by SEM and EDX analysis. The urchin-like WO_3_ particles acted as effective nucleating agents, increasing the crystallinity of PLA from 40% to 50% and achieving an impressive overall crystallinity rate of 97%. Differential scanning calorimetry (DSC) revealed an 11 K reduction in the crystalline phase transition temperature while maintaining stable melting (Tm) and glass transition (Tg) temperatures. Thermal analysis indicated a significant decrease in the onset of degradation and maximum thermal stability (T_max_), with a reduction of 21 K due to the incorporation of the WO_3_ particles. Optical measurements showed enhancement of UV-blocking properties from 9% to 55% with the WO_3_ particle loading. Rheological tests demonstrated substantial improvements in viscoelastic properties, including a remarkable 30-fold increase in storage modulus, suggesting enhanced gel formation. Although the nanocomposites showed minimal antibacterial activity against *Escherichia coli* and *Staphylococcus aureus*, they exhibited significant antifungal activity against *Candida albicans*. These results underscore the potential of the PLA/WO_3_ nanocomposites for advanced material applications, particularly where enhanced mechanical, thermal, optical, and antifungal performance is required.

## 1. Introduction 

Technological advancements have significantly increased the demand for novel materials with enhanced properties tailored to specific applications. One of the most active and promising areas of materials research is nanocomposite-based polymer matrices [1,2]. These materials offer numerous advantages, including low cost, lightweight, excellent chemical and mechanical resistance, and enhanced electrical conductivity [3]. Nanocomposites are synthesized by incorporating nanometric particles, either organic or inorganic, into a polymer matrix, which leads to enhanced properties due to the increased surface area and good dispersion within the matrix [4,5,6]. This dispersion plays a critical role in enhancing interactions between the matrix and the nanoparticles, leading to improved material properties [5,6]. The field of nanocomposites continues to grow as researchers explore innovative ways to improve material performance for diverse applications such as packaging, electronics, and biomedical devices, using the unique characteristics provided by nanoscale inclusions [3].

Among the various nanoparticles used in enhancing polymer matrices, tungsten trioxide (WO_3_) stands out due to its unique properties, including high density, chemical stability, and excellent optical characteristics, making it suitable for applications in sensors, optical devices, mechanical and X-ray shielding, and UV blocking [7,8]. The ability of WO_3_ to generate reactive oxygen species (ROS) under UV or visible light can be harnessed for antibacterial applications, making the composite useful in medical devices and implants. The incorporation of WO_3_ into polymer matrices has been extensively studied to improve the matrices’ performance [9,10,11,12,13]. Several studies have reported on WO_3_‘s integration into different polymers such as poly(aniline) [10,11], poly(3,4-ethylenedioxythiophene) [9], and PLA [12,13]. This research indicates that WO_3_ not only enhances the mechanical properties of these polymers but also introduces novel functionalities that can expand their application potential. For instance, the incorporation of WO_3_ can improve the thermal stability and UV resistance of the host polymer, thus extending its usability in harsh environmental conditions [11,13]. As such, WO_3_ nanocomposites represent a promising area for developing advanced materials that meet the increasing demands for high-performance applications.

PLA is one of the most commonly used biopolymers in the research domain and has gained significant attention in industries like food packaging due to its Generally Recognized As Safe (GRAS) status by the US Food and Drug Administration (FDA) [12,13,14]. PLA’s biodegradability and ease of processing make it an attractive alternative to conventional plastics derived from fossil fuels. As an aliphatic polyester polymer, PLA is primarily composed of polyglycolic or polyadic acid and is derived from renewable resources such as corn starch or sugarcane [14,15]. This biopolymer is not only environmentally friendly but also biocompatible, which has led to its application in medical fields such as prosthetics and dentistry, where it can replace synthetic polymers like polystyrene (PS) and polyethylene terephthalate (PET) [16,17]. Despite its advantages, PLA suffers from inherent drawbacks such as low thermal decomposition temperature, brittleness, and moisture absorption, which limit its widespread use [15]. To address these issues, researchers have explored the incorporation of various nanoparticles, including cellulose, ZnO, and TiO_2_, to enhance their mechanical, chemical, optical, and electrical properties [18,19,20,21].

Previous studies have demonstrated that honeycomb-like structures can enhance the mechanical and thermal properties of polymers [22,23,24], as seen in Qu et al.’s work on ZIF-67/PLA aerogels [22]. They observed that the nanofillers within the honeycomb-like pores could significantly adjust pore size and specific surface area, leading to improved material performance [22]. Applications of honeycomb-like morphologies span various fields, including water/oil separation, automotive engineering, and aerospace technology [22,25]. In this study, we aim to synthesize and characterize PLA/WO_3_ nanocomposites with a novel honeycomb-like morphology. The nanocomposites were prepared using a solution-casting method, incorporating WO_3_ particles with loadings up to 7 wt%. The use of WO_3_ is particularly intriguing due to its inherent biocompatibility, especially in controlled forms such as nanoparticles, thin films, or coatings, which makes it a promising candidate for biomedical applications when combined with PLA. Our study systematically explores the morphological development, structural processability, transparency, thermal, rheological, and antimicrobial properties of these nanocomposites. The results highlight the enhanced antifungal activity against *Candida albicans*, suggesting that these PLA/WO_3_ nanocomposites offer a viable approach for use in a wide range of applications, potentially extending into industries such as environmental technology. The research not only showcases the potential of the honeycomb-like structure in improving polymer properties but also opens new avenues for advanced material applications.

## 2. Experimental 

### 2.1. Chemicals and Reagents

For the preparation of PLA/WO_3_ nanocomposite films, high-purity chemicals and reagents were used. PLA, sourced from NETCO EXTRUDED PLASTICS, (Hudson, MA, USA), with a purity exceeding 98%, served as the polymer matrix. Dichloromethane (CH_2_Cl_2_, ≥99.9%, Riedel-de Haën, Buchs, Switzerland) was used as the solvent to dissolve PLA, facilitating the creation of a homogeneous solution. Tungsten oxide powder (urchin-like WO_3_ particles, 99% purity, Sigma Aldrich, Saint Louis, MI, USA) was employed as the nanofiller. For its dispersion, a mixture of ethanol (C_2_H_5_OH, 96.96%, GPR RECTAPUR, VWR, Radnor, PA, USA) and distilled water was used, ensuring effective nanoparticle dispersion.

### 2.2. Preparation of PLA/WO_3_ Nanocomposite

The PLA/WO_3_ nanocomposite films were prepared using a solution-casting method. Initially, 2 g of PLA were dissolved in 50 mL of dichloromethane (CH_2_Cl_2_). The dissolution was carried out under constant stirring at 50 °C for 15 min to ensure complete dissolution of the PLA and to achieve a viscous solution. Once the PLA was fully dissolved, 0.5 g of WO_3_ particles were dispersed in a mixture of 25 mL ethanol and 25 mL distilled water with sonication for 1 h to ensure complete dispersion of the WO_3_ particles. The dispersed WO_3_ particles were then added at weight percentages of 1%, 3%, 5%, and 7% relative to the weight of the PLA and mixed at 45 °C for 4 h (Table 1). FTIR spectroscopy confirmed the complete evaporation of the solvent from the films, as no residual solvent peaks were observed, ensuring the successful formation of solid nanocomposite films.

### 2.3. Physicochemical Characterization 

A series of characterization techniques were employed to analyze the structural and morphological properties of the prepared PLA/WO_3_ nanocomposites. X-ray diffraction (XRD) measurements were performed using a Bruker D2 Phaser diffractometer with Cu Kα radiation at 30 kV and 10 mA. The XRD patterns were recorded over a 2θ range from 2° to 80° with a scan step size of 0.01°/s to determine the crystallinity and phase composition of the nanoparticles and nanocomposites. The average crystallite size of the WO_3_ nanoparticles was calculated using the Debye–Scherrer equation [26].

Scanning Electron Microscopy (Quanta 650, FEI, Hillsboro, OR, USA) operating at an acceleration voltage of 10 kV was employed to analyze the particle size and morphology of WO_3_ powder, pure PLA after solution casting, and PLA/WO_3_ nanocomposites. The WO_3_ powder was directly mounted on the SEM sample holder to capture its native morphology without additional treatment. For the PLA and PLA/WO_3_ nanocomposites, the films were spread on petri dishes, dried completely, and stored at −10 °C for 12 h to facilitate cryofracturing, ensuring clean cross-sectional surfaces, which is essential for polymer composites with irregular surfaces. Following cryofracturing, the samples were mounted for SEM analysis, with a focus on regions that best represented the material’s morphology. Elemental analysis was performed using an energy-dispersive spectrometer (EDS) attached to the SEM, providing information on the distribution of WO_3_ particles within the PLA matrix. Pore size distribution was determined using Image J software 1.49.

Fourier transform infrared spectroscopy (FTIR) was used to identify the functional groups within the nanocomposites. FTIR spectra were collected using a JASCO FT/IR-4100 spectrometer (Easton, MD, USA) equipped with an ATR (attenuated total reflectance) accessory, covering the frequency range of 400–4000 cm^−1^ at a resolution of 4 cm^−1^. Data were acquired at a scan rate of 2 scans per second and averaged over 64 scans to enhance signal-to-noise ratio and ensure high-quality analysis.

The UV-visible-NIR spectroscopic analysis of the polymer films was conducted using a Shimadzu UV-1201PC spectrometer (Shimadzu, Kyoto, Japan), covering the wavelength range from 200 to 800 nm. The films were prepared on quartz substrates to avoid interference. The scan speed was set to 200 nm/min, with a 1 nm interval for high-resolution data acquisition. This analysis was performed to evaluate the UV-blocking properties of the films, focusing on their absorbance in the UV region (200–400 nm) to assess their effectiveness in blocking ultraviolet light. Additionally, the bandgap energy of the films was calculated using the Tauc plot method by plotting (αhν)^2^ vs. photon energy (hν), where α is the absorption coefficient. The bandgap was determined by extrapolating the linear portion of the curve to the energy axis, providing insight into the electronic properties of the polymer nanocomposites. This method is crucial for understanding how the WO_3_ particles influence the optical characteristics and electronic interactions within the composite matrix.

The film thickness was measured using a digital micrometer with a precision of ±0.005 mm at ten random locations per film. Measurements were taken gently to avoid deformation, and the average thickness was calculated along with the standard deviation to account for variations. This ensures accurate interpretation of UV-Vis absorbance data.

### 2.4. Rheological Measurements

Dynamic rheological measurements were conducted using a cone-plate rheometer (MCR302, Anton Paar GmbH, Graz, Austria) to evaluate the viscoelastic properties of PLA/WO_3_ nanocomposite gels before solvent evaporation. The samples, prepared by mixing PLA and WO_3_ in solution to form a gel, were loaded onto the 60 mm diameter cone (1° angle) with a gap set at 0.125 µm to minimize edge effects. Care was taken to avoid air bubbles during loading, and a solvent trap was employed to prevent solvent evaporation. A dynamic strain sweep (0.01% to 100% strain) at 1 Hz was performed to determine the linear viscoelastic region (LVR), followed by frequency sweep tests (0.01 Hz to 100 Hz) conducted at 0.1% strain within the LVR. All measurements were performed at 37 °C using a Peltier heating system to ensure temperature control. The storage modulus (G′), loss modulus (G″), and complex viscosity (η*) were recorded, with each sample tested in triplicate for reproducibility. The method, including careful sample handling, gap setting, and solvent management, ensures the reliability and reproducibility of the rheological data.

### 2.5. Thermal Analysis 

Thermogravimetric analysis (TGA) was performed using a Q500 instrument (TA Instruments, New Castle, DE, USA) to evaluate the thermal stability of the films. Approximately 5 mg of each sample was heated from ambient temperature to 700 °C at a rate of 10 °C/min under a nitrogen flow of 60 mL/min. Differential scanning calorimetry (DSC) was conducted with a TA Instruments DSC Q20 (New Castle, DE, USA) to analyze the thermal transitions of the PLA/WO_3_ nanocomposites. Samples weighing 5 mg were heated from room temperature to 200 °C at 10 °C/min, then cooled back to 20 °C at the same rate while maintaining a nitrogen flow of 40 mL/min. DSC thermograms provided data on crystallization and melting behavior. Crystallization and melting enthalpies were determined using the theoretical maximum melting enthalpy $\Delta H_{m}^{0}=93.6\;J/g$ [27]. The relative degree of crystallization rate was calculated by Equation (1) [28].

(1) $$\chi \;\left(\%\right)=\frac{\Delta H_{m}}{\left(1-Wt_{f}\right)\cdot \Delta H_{m}^{0}}$$

where Δ*H_m_* represents the measured melting enthalpy and *Wt_f_* is the weight fraction of WO_3_ particles in the nanocomposite [28].

### 2.6. Antimicrobial Assays 

The antimicrobial activity of WO_3_ particles and PLA/WO_3_ nanocomposites was evaluated using the agar disc diffusion method [29,30]. Testing was conducted against *Staphylococcus aureus* ATCC 25923 (Gram-positive bacteria), *Escherichia coli* ATCC 25922 (Gram-negative bacteria), and *Candida albicans* ATCC 10231 (fungal strain). Microbial cultures were prepared fresh: the fungus was grown on Sabouraud agar at 25 °C for 48 h, and bacterial strains on nutrient agar at 37 °C for 24 h. Bacterial cultures were adjusted to a density of 10^6^ CFU/mL [31]. For the assay, 5 mL of brain heart infusion broth (BHIB) was mixed with 0.1 g of PLA/WO_3_ nanocomposites, PLA, and WO_3_ particles. Net PLA film (0.1 g) and 100 µL of a 100 µg/mL WO_3_ particle suspension were used as controls. Suspensions were spread on nutrient and Sabouraud agar plates, and sterile discs (6 mm) soaked in the solutions were placed on the agar using sterile forceps. Plates were incubated at 37 °C for 24 h for bacterial strains and at 25 °C for 48 h for the fungal strain. Antimicrobial activity was assessed by measuring the zone of inhibition around the discs, with all tests performed in duplicate for accuracy.

## 3. Results and Discussions

### 3.1. Analysis of WO_3_ Nanoparticles

**Figure 1:**
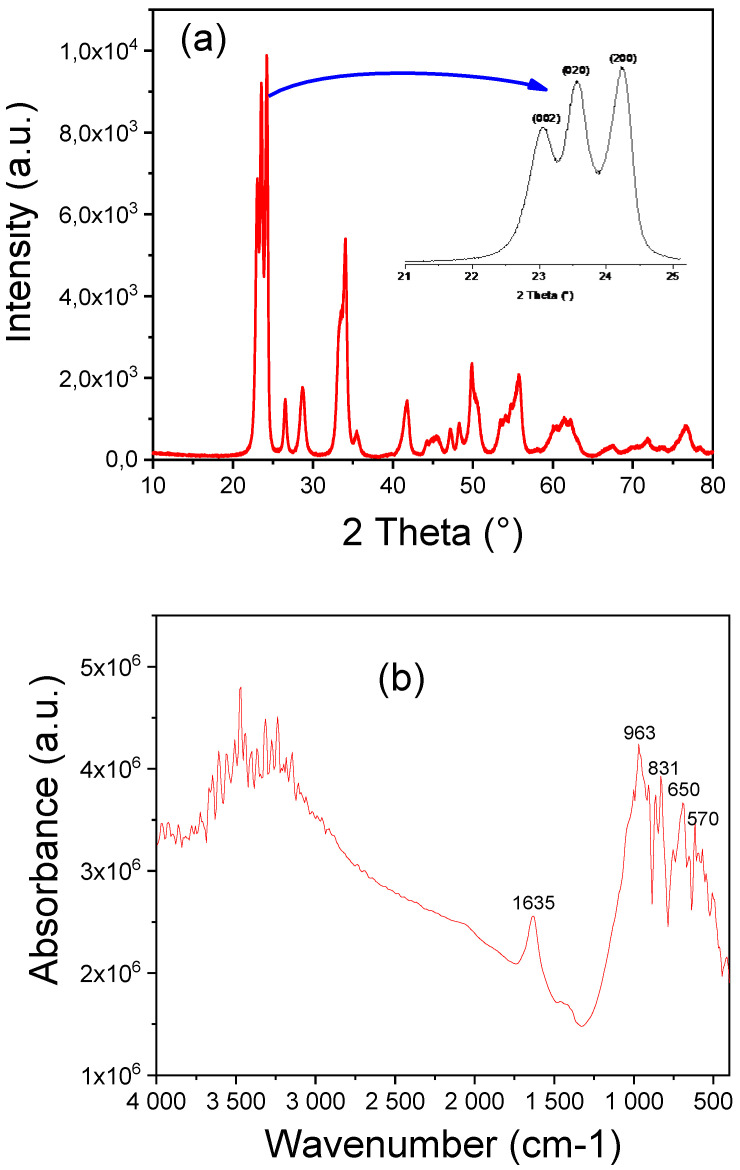
Characteristics of the urchin-like WO_3_ particles: (**a**) XRD patterns; (**b**) FTIR spectra.

The XRD pattern of the WO_3_ particles, shown in Figure 1a, reveals a series of well-defined peaks at 2θ values of 23.06°, 23.56°, 24.20°, 26.55°, 28.72°, 34.11°, 35.51°, 41.78°, 47.20°, 49.88°, and 55.70°. These peaks correspond to the (002), (020), (200), (120), (112), (022), (202), (122), (222), (320), (040), (440), and (620) planes of monoclinic WO_3_ [32,33], as per JCPDS No. 71-0131. The presence of these sharp peaks confirms the high crystallinity of the WO_3_ particles. Notably, the absence of any additional peaks in the XRD pattern indicates the formation of a pure WO_3_ phase, free from impurities or secondary phases. By analyzing the most intense peak at 2θ = 24.20° (the (200) plane), the average crystallite size of the WO_3_ particles was determined to be 20.7 nm, with a crystallinity of 96.9%.

Figure 1b presents the Fourier-transform infrared (FTIR) spectra of WO_3_ particles. The FTIR spectra of WO_3_ particles can be divided into three distinct zones: a broad band at 3200–3600 cm^−1^, a peak at 1635 cm^−1^, and a third zone comprising narrow peaks between 570 and 960 cm^−1^. The bands at around 3467 cm^−1^ are attributed to the O-H stretching mode due to adsorbed water molecules. The peak at 1635 cm^−1^ is associated with the H-O-H bending vibration mode [33]. The band at 963 cm^−1^ corresponds to the asymmetric stretching vibrations of W=O bonds [34,35]. Furthermore, the narrow peaks between 650 and 831 cm^−1^ are assigned to the (W-O-W) and (O-W-O) stretching vibrations of bridging oxygen and tungsten, respectively [11,35].

**Figure 2:**
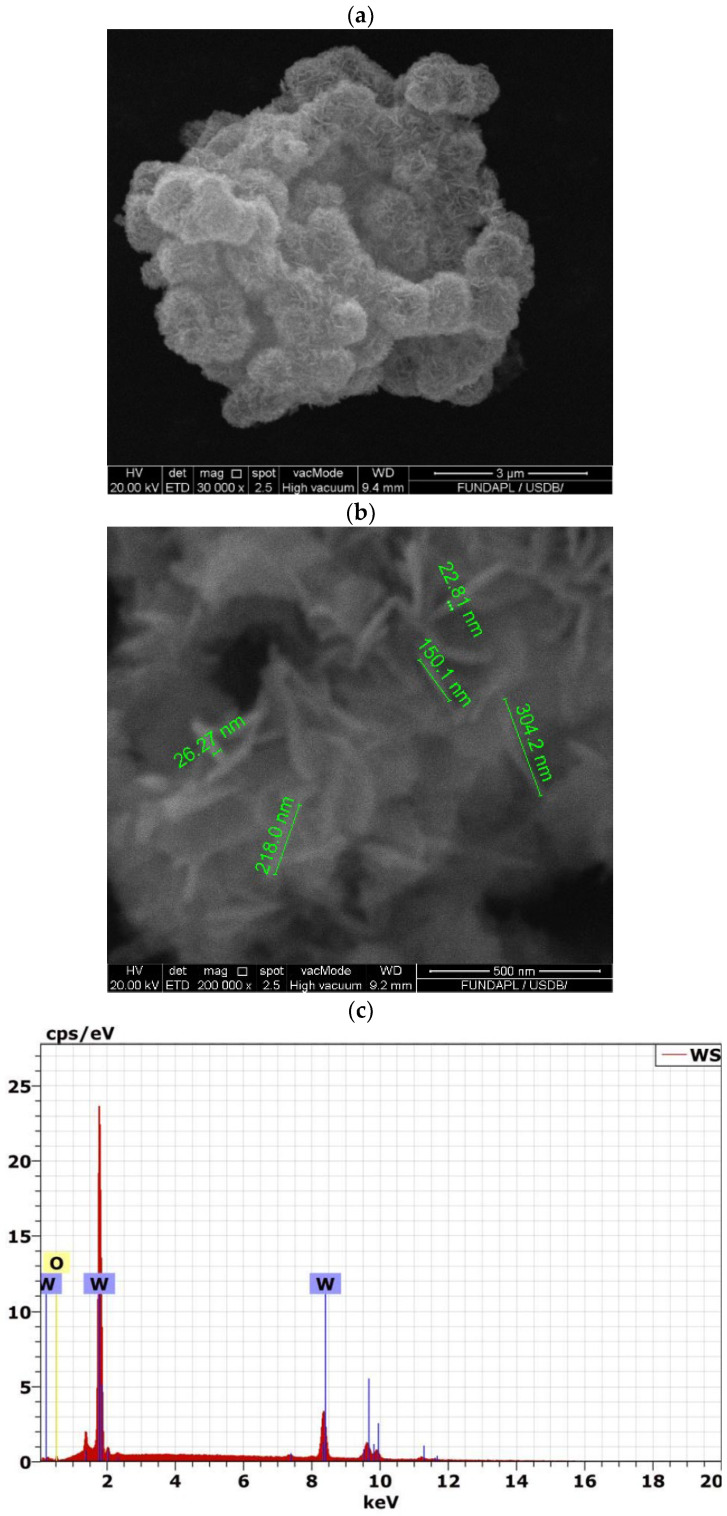
SEM analysis of the urchin-like WO_3_ particles: (**a**,**b**) SEM images; (**c**) EDX-SEM analysis.

The morphology of the WO_3_ particles, as shown in Figure 2a,b, features an intriguing structure. The WO_3_ powder consists of compact agglomerations of urchin-like particles approximately 0.5 µm in diameter, similar to results observed in [36]. These aggregates are composed of densely packed nanorods with an average width of 24 nm and a shape factor (L/d ratio) of 8. This distinctive nanorod morphology significantly increases the surface area, enhancing interaction with the surrounding polymer matrix. This unique structure is crucial in improving the mechanical properties of polymer composites, particularly in applications where enhanced performance is required [37].

The SEM-EDX analysis of the WO_3_ nanoparticles, shown in Figure 2c, further substantiates the high purity of the sample. The EDX spectrum displayed prominent peaks at 1.7 keV and 8.4 keV for tungsten (W) and a peak at 0.5 keV for oxygen (O). Quantitative analysis revealed that tungsten constituted 75.3% by weight and oxygen constituted 24.7% by weight, closely matching the theoretical stoichiometry of WO_3_. This result confirms the purity of the WO_3_ particles, with no extraneous elements detected, emphasizing the absence of impurities. Such purity is crucial for ensuring the integrity and performance of PLA/WO_3_ nanocomposites, as it allows for optimal interaction between the nanofillers and the polymer matrix, ultimately contributing to enhanced structural and functional properties.

### 3.2. Crystallinity and Polymorph of PLA/WO_3_ Nanocomposites

**Figure 3:**
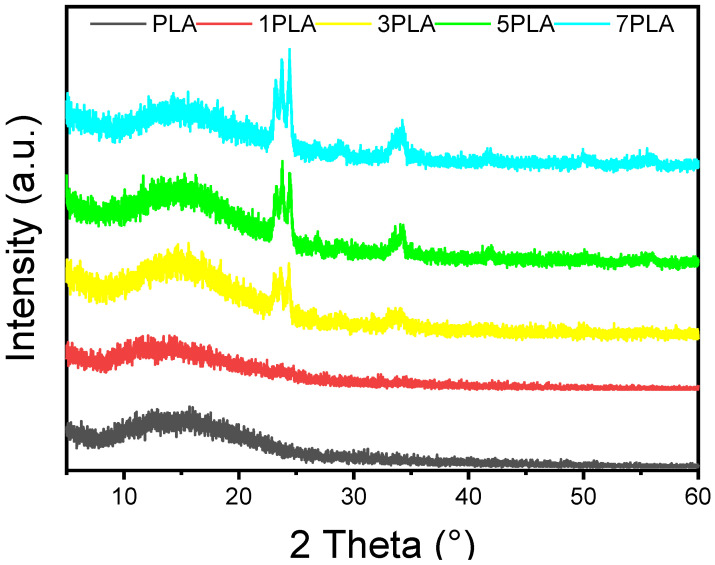
XRD spectra of neat PLA and PLA/WO_3_ nanocomposites loaded with different loadings of the urchin-like WO_3_ particles (1 wt%, 3 wt%, 5 wt%, and 7 wt%).

Figure 3 displays the XRD patterns of neat PLA and PLA/WO_3_ nanocomposites with varying loadings of WO_3_ particles. The XRD pattern of pure PLA shows a broad amorphous peak centered at 2θ = 15.3°, along with a barely detectable crystalline peak at 2θ = 31.7°, underscoring the semi-crystalline nature of PLA [38,39]. The broad amorphous peak is attributed to disordered regions in the PLA polymer chains, while the crystalline peak represents ordered regions. The XRD patterns of PLA/WO_3_ nanocomposites exhibit PLA peaks along with the most intense peaks of WO_3_ particles at 2θ values of 23.06°, 23.56°, 24.20°, 26.55°, 34.11°, and 35.51° corresponding to the (002), (020), (200), (120), and (022), (202) planes of monoclinic WO_3_, as per JCPDS No. 71-0131. While the incorporation of nanoparticles facilitates nucleation and improves alignment of polymer chains, leading to a slight increase in the intensity of the PLA crystalline peaks, this change in crystallinity is minimal. The urchin-like WO_3_ particles act as nucleation sites, but their impact on overall PLA crystallinity is not significant. This finding is consistent with observations in similar polymer-filler systems [39,40].

### 3.3. Morphological Structure of PLA/WO_3_ Nanocomposites

**Figure 4:**
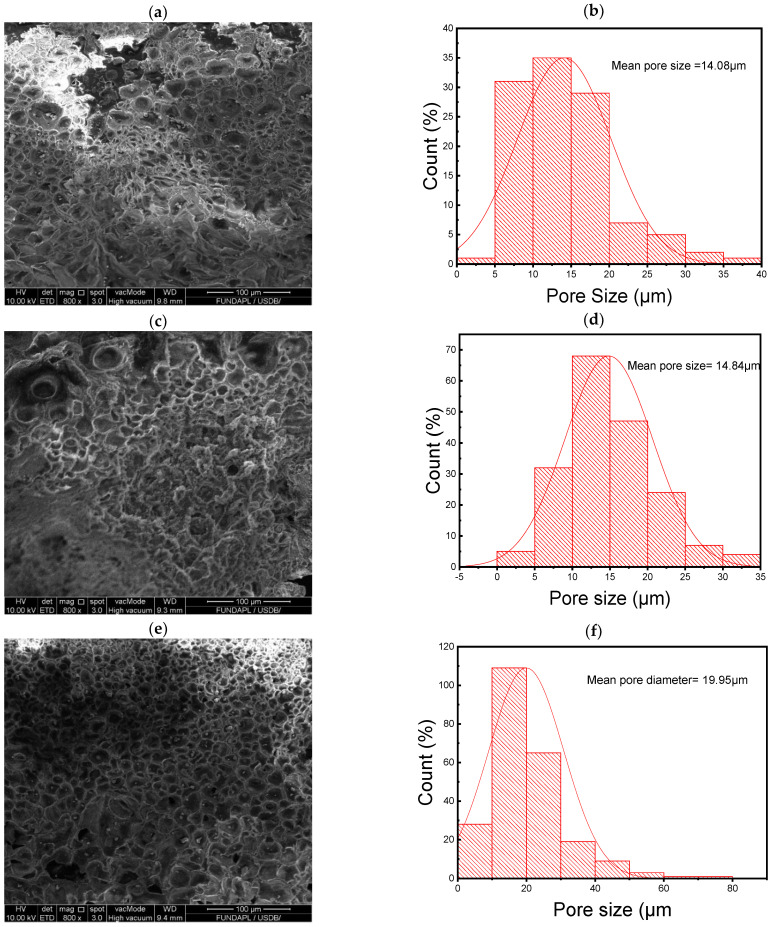

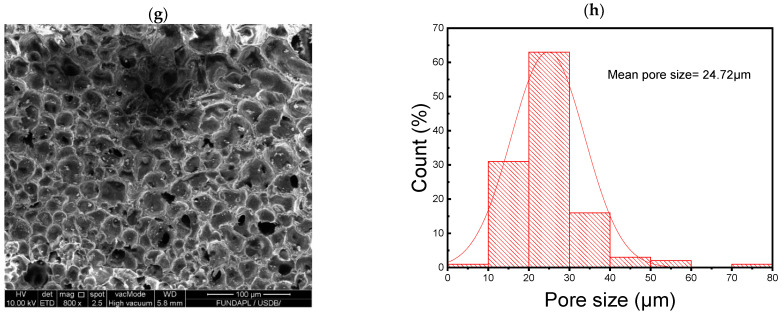
SEM cross-section images illustrating the irregular honeycomb-like morphology of PLA/WO_3_ nanocomposites with different urchin-like WO_3_ particle loading with pore size distribution: (**a**,**b**) 1 wt%, (**c**,**d**) 3 wt%, (**e**,**f**) 5 wt%, and (**g**,**h**) 7 wt%.

**Figure 5:**
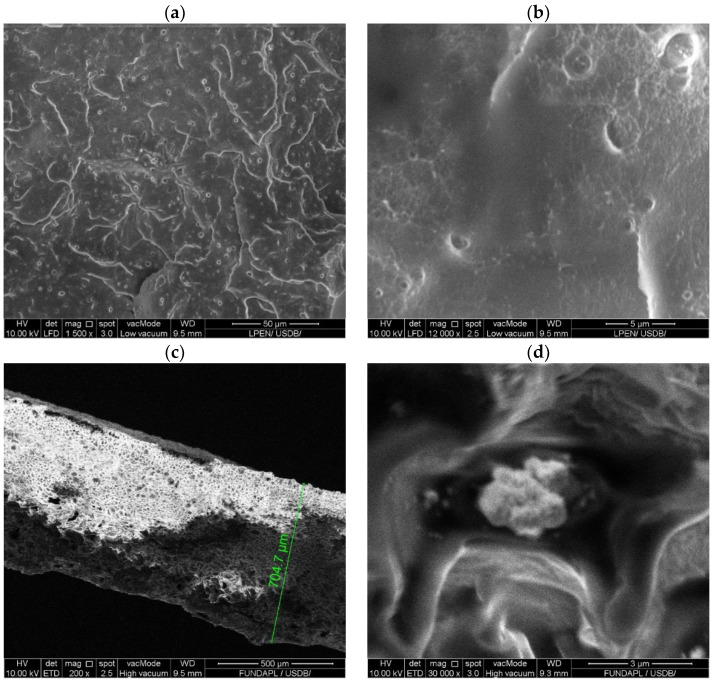
SEM cross-section images illustrating: (**a**,**b**) the smooth surface of pure PLA; (**c**,**d**) the irregular honeycomb-like morphology of PLA/WO_3_ nanocomposites with 3 wt% urchin-like WO_3_ particle loading.

Figure 4 illustrates the structural evolution of PLA/WO_3_ nanocomposites with varying WO_3_ particle loadings. SEM cross-sectional images in Figure 4 reveal a honeycomb-like morphology across all samples, which becomes more pronounced as the WO_3_ content increases from 1 wt% to 7 wt%, causing the average pore size to expand from 14.8 µm to 24.92 µm and the pores to become more circular (Figure 4b,d,f,h) and (Table 1). This indicates that the WO_3_ particles act as structural modifiers, disrupting the polymer chains and creating larger voids within the matrix. Higher magnification images further show that the urchin-like WO_3_ particles tend to cluster within these pores, with more prominent agglomeration at higher concentrations. These morphological changes have significant implications for the nanocomposites’ mechanical properties, such as enhanced tensile strength and elasticity, while the increased porosity and surface area make them ideal for applications in filtration and catalysis. Figure 5 complements this by comparing Net PLA after solution casting with PLA/WO_3_ nanocomposites containing 3 wt% WO_3_ particles. The pure PLA exhibits a smooth surface, while the addition of WO_3_ particles introduces a honeycomb-like structure, consistent with higher WO_3_ loadings. The WO_3_ particles disrupt the polymer matrix, forming larger voids, and at higher magnifications, they are seen clustering within the pores, likely due to surface energy minimization. The uniform pore size and strong interactions between the PLA matrix and WO_3_ particles result in a pore network, similar to trends observed in ZIF-67 nanofillers, where increased filler concentration leads to structural modification and pore expansion [22].

### 3.4. Chemical Bonding in PLA/WO_3_ Nanocomposites

**Figure 6:**
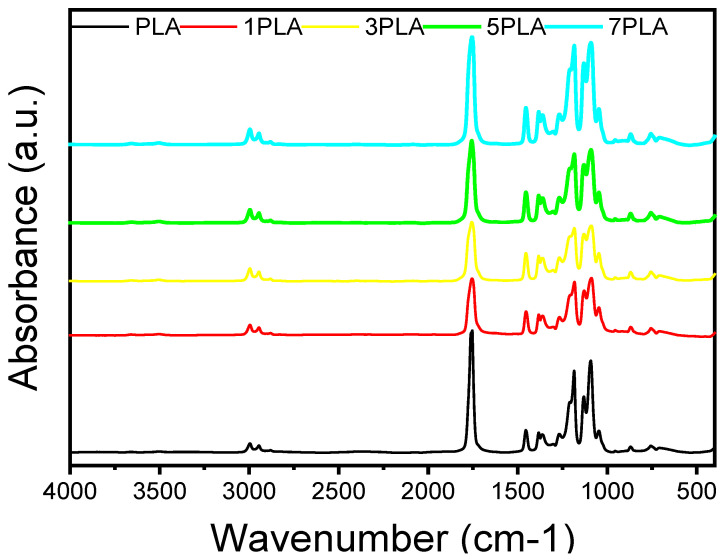
FTIR spectrum of PLA/WO_3_ nanocomposites with varying urchin-like WO_3_ particle loading (0 wt%, 1 wt%, 3 wt%, 5 wt%, and 7 wt%).

Figure 6 presents the FTIR spectra of PLA and PLA/WO_3_ nanocomposites, highlighting the interaction between the polymer matrix and WO_3_ particles. The FTIR spectrum of neat PLA shows characteristic peaks at 2939 cm^−1^ and 2999 cm^−1^, corresponding to the C-H stretching vibrations of asymmetric and symmetric modes in -CH_3_ and -CH groups [28]. Additional bands appear at 1454 cm^−1^ for CH_3_ stretching, 1362 cm^−1^ for C-H deformation, 1185 cm^−1^ for C-O stretching, 1089 cm^−1^ for O-C=O stretching, and a sharp peak at 1757 cm^−1^ for C=O stretching [41,42]. In the PLA/WO_3_ nanocomposites, no new peaks are observed, indicating that no new chemical bonds are formed between the PLA matrix and the urchin-like WO_3_ particles. This lack of new peaks suggests that interactions between PLA and the urchin-like WO_3_ particles are primarily physical rather than chemical [27,43,44]. Despite the absence of new chemical bonding, the spectra show increased intensity of existing absorption bands, indicating effective incorporation of the urchin-like WO_3_ particles into the PLA matrix. This incorporation enhances the matrix’s density and uniformity [45]. The slight shifts in absorption peaks may reflect interactions affecting PLA chain mobility, but the lack of stronger chemical bonding limits potential improvements in thermal and barrier properties [46].

### 3.5. UV-Absorbance and Blocking of PLA/WO_3_ Nanocomposites

**Figure 7:**
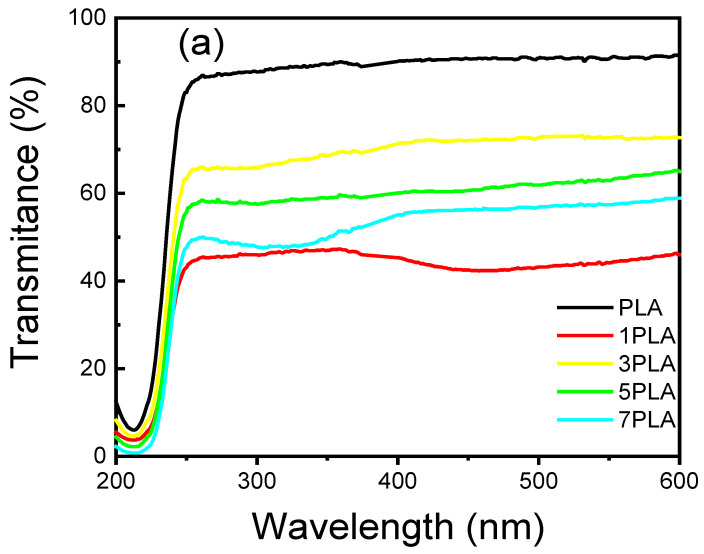

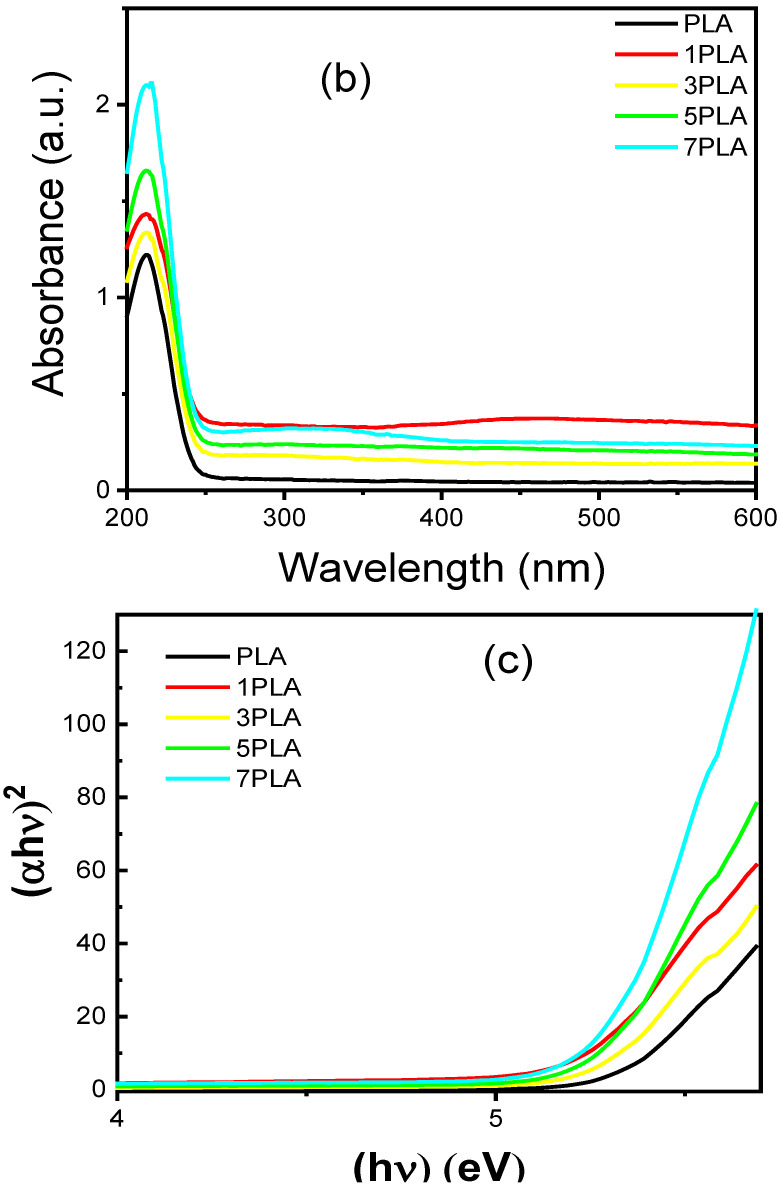
UV-Vis spectra of PLA and PLA/WO_3_ nanocomposites. (**a**) Transmittance, (**b**) absorbance, and (**c**) energy band gap.

The UV-absorbance and UV-blocking capabilities of PLA/WO_3_ nanocomposites are essential for applications where light sensitivity can compromise the shelf life and quality of products [47]. The incorporation of the urchin-like WO_3_ particles into the PLA matrix significantly modifies the optical properties of the nanocomposites, as shown in Figure 7a. With the addition of WO_3_ particles, the UV-Vis transmittance of the nanocomposites decreases notably compared to neat PLA. This reduction is primarily due to the enhanced light scattering and absorption provided by the WO_3_ particles, which results in reduced transparency. At a wavelength of 600 nm, neat PLA exhibits a transmittance of 91%, whereas the 1 wt% PLA/WO_3_ nanocomposite (1PLA) shows a significantly reduced transmittance of 45%. This dramatic decrease indicates an effective distribution of the urchin-like WO_3_ particles within the polymer matrix, optimizing light-blocking efficiency. For the other composites, the transmittance values are 72% for 3PLA, 62% for 5PLA, and 58% for 7PLA, demonstrating an upward trend in UV-blocking capabilities with increased nanoparticle concentration (Table 1). This behavior can be attributed to the structural properties of the nanocomposites, including pore size and circularity, which influence the interaction between the matrix and incident light. The 1PLA sample, for instance, has a pore length of 14.01 µm, a pore width of 11.11 µm, and a pore circularity of 1.26, which likely contributes to its optimal light-blocking performance.

Figure 7b further illustrates that the introduction of the urchin-like WO_3_ particles substantially increases absorbance across the visible and UV ranges. The 1PLA sample exhibits a remarkable 45% increase in absorbance compared to neat PLA, underscoring its potential for protecting light-sensitive packaged products. The absorbance spectra reveal a significant rise in the UV region, particularly between 200 and 250 nm, which highlights the nanocomposites’ improved UV-blocking capabilities. This is likely due to the π-π* transitions in the ester groups of PLA, with the presence of WO_3_ particles altering electronic interactions within the matrix [48]. As shown in Figure 7c, the bandgap energies decrease with increasing the urchin-like WO_3_ particle loading: 5.39 eV for PLA, 5.15 eV for 1PLA, 5.21 eV for 3PLA, 5.22 eV for 5PLA, and 5.21 eV for 7PLA. The reduction of 0.2 eV observed for the 7 wt% WO_3_ particles suggests enhanced electronic coupling within the composite. This aligns with findings from Shanshool et al. [49], who reported similar reductions in bandgap energies in various polymer matrices with increased nanofiller concentrations. The optical characteristics of PLA/WO_3_ nanocomposites, particularly their UV-blocking efficiency, make them promising candidates for applications requiring improved light protection and thermal stability. The data presented in Table 1 reinforce the importance of the urchin-like WO_3_ particle loadings and distribution in achieving desired optical properties, offering a pathway for tailoring nanocomposites to specific industrial needs.

### 3.6. Thermal Stability of PLA/WO_3_ Nanocomposites

**Figure 8:**
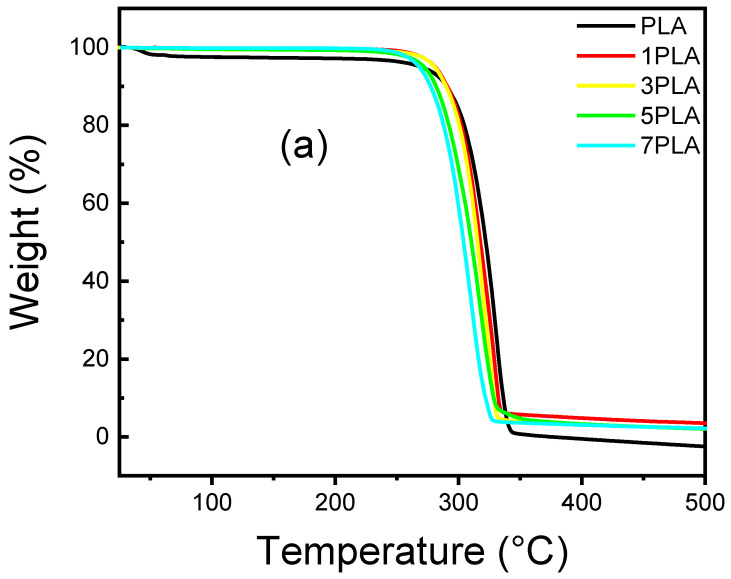

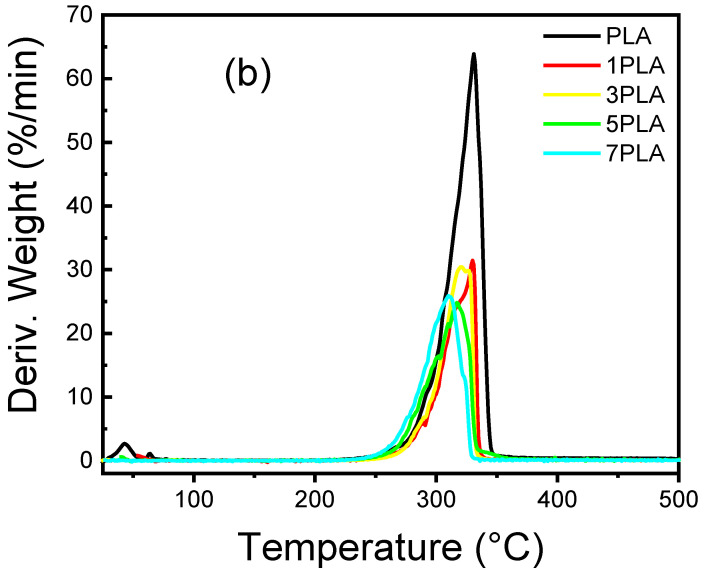
TGA curves of the neat PLA and PLA/WO_3_ nanocomposites: (**a**) Weight loss curves illustrating the thermal degradation profiles, and (**b**) derivative TGA (d-TGA) graphs showing the rate of weight loss as a function of temperature.

TGA analysis was used to evaluate the thermal stability of neat PLA and PLA/WO_3_ nanocomposites, as shown in Figure 8 and Table 2. Neat PLA exhibited an initial weight loss of 4 wt% between 40 and 120 °C, which was higher compared to PLA/WO_3_ nanocomposites (1.5 wt%). This reduction in early weight loss in the nanocomposites can be attributed to the porous structure facilitating more efficient solvent removal. The initial weight loss in neat PLA is likely due to residual solvents from the casting process or absorbed moisture [50]. Incorporating urchin-like WO_3_ particles significantly enhances the thermal stability of the PLA nanocomposites, with the WO_3_ particles functioning as reinforcing agents that limit polymer chain mobility and act as thermal barriers, thereby slowing heat transfer and reducing the risk of thermal degradation [27,43]. Increased WO_3_ particle loadings (1–5 wt%) raise the temperature at which 5%, 50%, and 95% weight loss occurs, demonstrating enhanced thermal resistance. For example, the T5% for neat PLA is 269 °C, while it rises to 281–282 °C for 1–5 wt% WO_3_ nanocomposites, indicating a clear improvement in thermal stability. However, at 7 wt% WO_3_, T_5%_ decreases slightly to 268 °C, suggesting that excessive WO_3_ may compromise thermal stability. This trend is similarly reflected in the T_50%_ values, which decrease from 322 °C for neat PLA to 304 °C for 7 wt% WO_3_, indicating that higher WO_3_ loadings may catalyze degradation by introducing defects through aggregation. The final degradation temperature (T_95%_) and maximum degradation rate (T_max_) also show this pattern, with T_95%_ increasing to 389 °C for 1 wt% WO_3_ but dropping to 326 °C for 7 wt% WO_3_. Likewise, T_max_ decreases from 331 °C for neat PLA to 310.4 °C for 7 wt% WO_3_. These findings suggest that while lower WO_3_ concentrations improve thermal stability, higher concentrations may initiate degradation pathways, consistent with previous findings on ZnO nanofillers in PLA by Murariu et al. [51].

### 3.7. DSC Analysis of PLA/WO_3_ Nanocomposites

**Figure 9:**
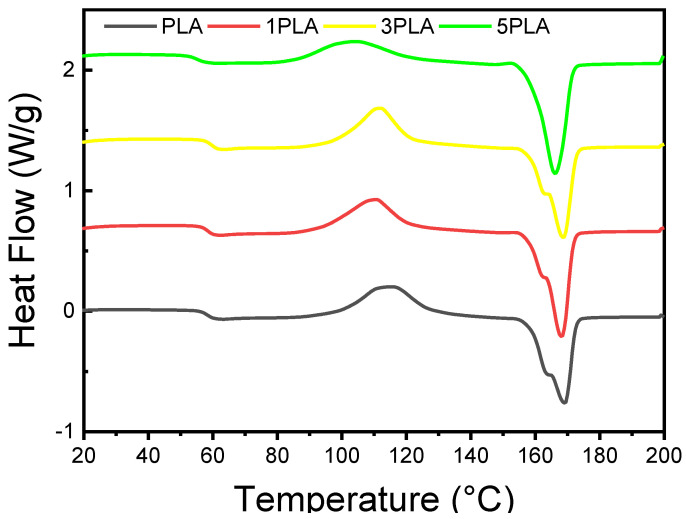
DSC scan of neat PLA and PLA/WO_3_ composites with different amounts of WO_3_ (1, 3, and 5 wt%).

DSC was used to assess the impact of urchin-like WO_3_ particles on the thermal properties of PLA/WO_3_ nanocomposites, focusing on key parameters such as glass transition temperature (Tg), cold crystallization temperature (Tc), melting temperatures (T_m1_ and T_m2_), and crystallization rate (χ_c_). The DSC thermograms, presented in Figure 9, indicate that the addition of WO_3_ particles leads to significant changes in these thermal properties. For neat PLA, the DSC profile and Table 3 show a Tg of 58 °C, a Tc of 115 °C, and two melting temperatures (T_m1_ at 164 °C and T_m2_ at 169 °C), suggesting the presence of different crystalline phases within the polymer matrix [52,53,54]. When 1 wt% WO_3_ (1PLA) is loaded, Tg increases slightly to 59 °C, indicating a minor plasticizing effect, and Tc shifts to 112 °C. The melting temperatures slightly decrease to 163 °C for Tm1 and 168 °C for T_m2_. These changes suggest that the WO_3_ particles are affecting the thermal properties by promoting better nucleation and modifying the crystallization behavior.

At higher WO_3_ concentrations, further alterations are observed. For the 3 wt% WO_3_ (3PLA) sample, Tg increases to 58 °C, while Tc decreases to 110 °C, and the melting temperatures adjust to 162 °C (T_m1_) and 168 °C (T_m2_) (Table 3). The crystallization rate (χ_c_) rises to 42.86%, reflecting enhanced nucleation effects due to WO_3_ particles. In contrast, for the 5 wt% WO_3_ (5PLA) nanocomposites, Tg decreases to 55 °C, and Tc further decreases to 104 °C. The melting shoulder (T_m1_) disappears, indicating a more homogeneous melting process, with the remaining melting temperature (T_m2_) at 166 °C [55,56]. The crystallization rate peaks at 50.53%, demonstrating effective nucleation and higher crystallinity. These results underscore the significant role of WO_3_ particles in altering PLA’s thermal properties, enhancing nucleation, and modifying both glass transition and melting behaviors.

### 3.8. Rheological Properties of PLA/WO_3_ Nanocomposites

**Figure 10:**
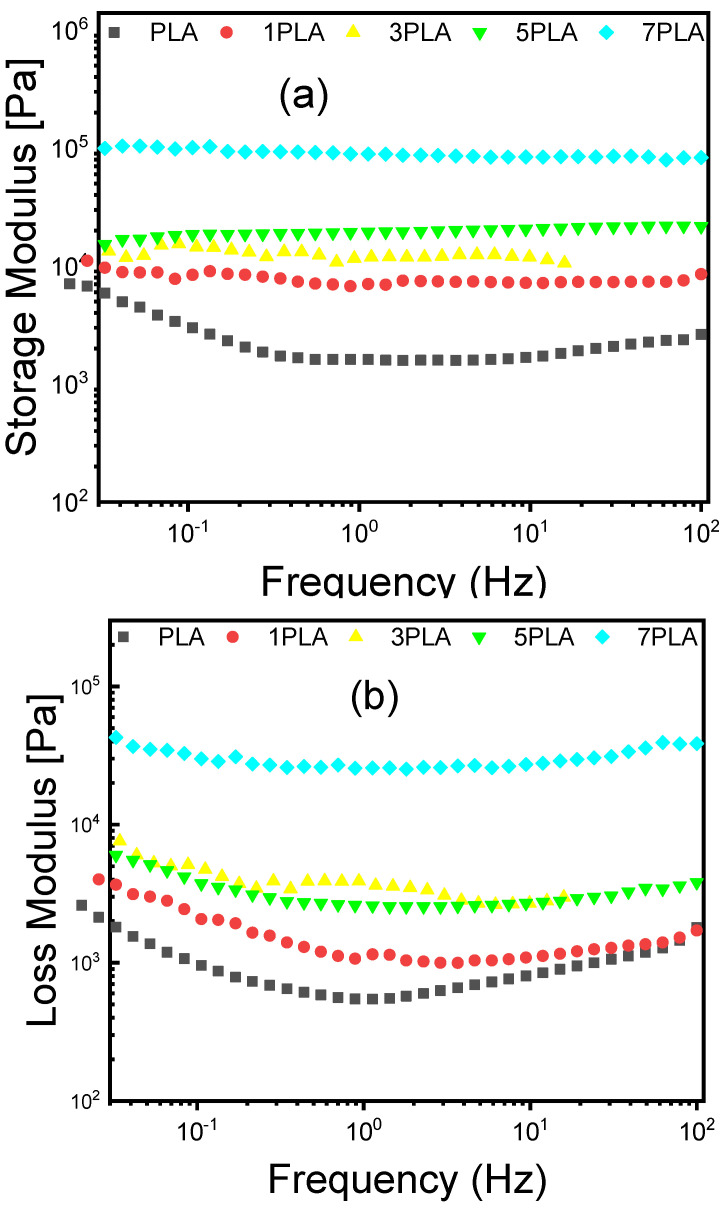

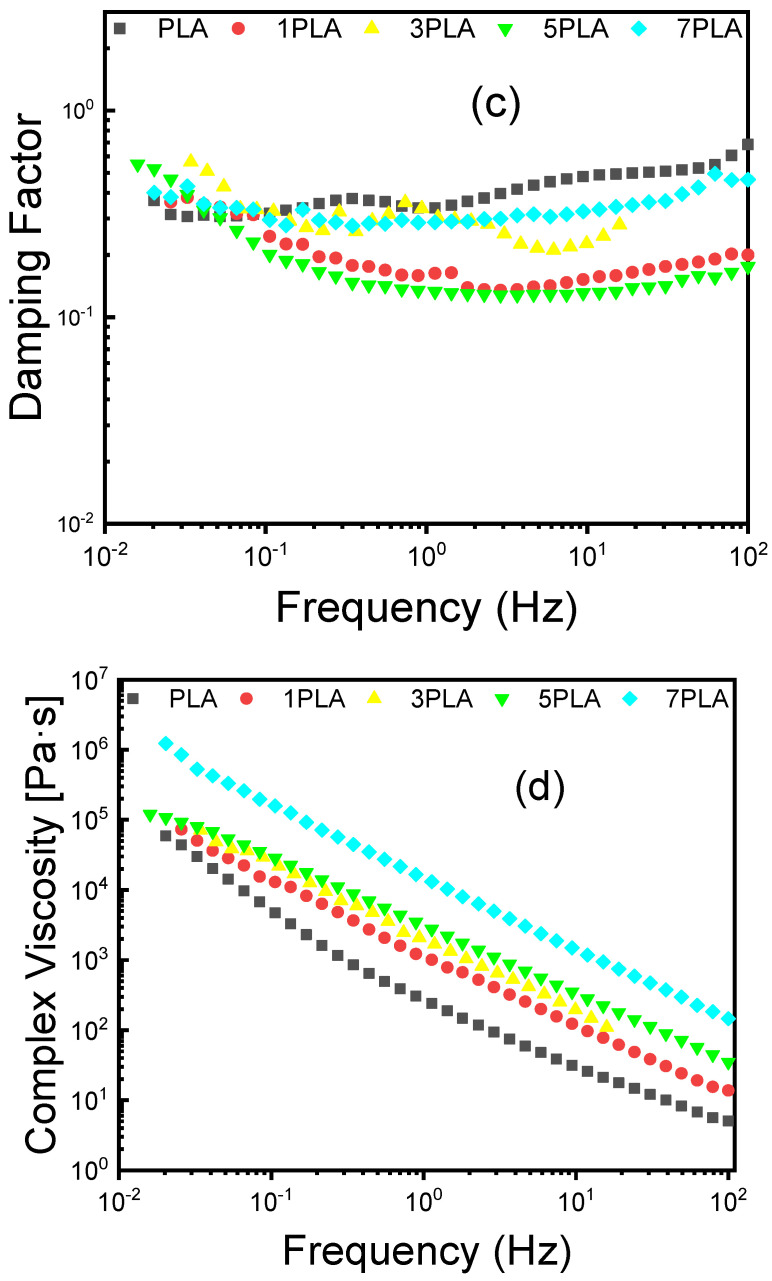
Variation of storage, loss moduli, damping factor, and complex viscosity in function of the frequency of neat PLA and PLA/WO_3_ nanocomposites: (**a**) storage modulus; (**b**) loss modulus; (**c**) damping factor; (**d**) complex viscosity.

Rheological properties are crucial for understanding the processing and performance of nanocomposites. The effects of WO_3_ particles on PLA were assessed through frequency- and temperature-dependent rheological tests. The results, shown in Figure 10, provide insights into the storage modulus (G′), loss modulus (G″), damping factor (tan δ), and complex viscosity.

Figure 10a,b illustrate that both G′ and G″ exhibit minimal frequency dependence over a broad range of frequencies, indicating that the nanocomposites form a well-structured 3D network [57]. For neat PLA, G′ is equal to 8.42 × 10^3^ Pa, which increases to 1.01 × 10^5^ Pa at 7 wt% WO_3_. This substantial increase in G′ with higher WO_3_ particle loading suggests that the nanostructured particles enhance the gel network’s rigidity and strength. The higher storage modulus signifies that the PLA/WO_3_ nanocomposites have improved mechanical stability and resistance to deformation. This enhancement can be attributed to the effective reinforcement provided by the WO_3_ particles, which interact strongly with the PLA matrix and improve its structural integrity.

In contrast, the loss modulus (G″), which represents the energy dissipation due to viscous effects, also increases with the concentration of WO_3_ particles (Figure 10c). For 1PLA sample, G″ is 2.07 × 10^3^ Pa, whereas it rises to 2.99 × 10^4^ Pa for 7PLA. This increase in G″ reflects higher frictional forces and energy dissipation within the material as more WO_3_ particles are introduced [58,59]. Enhanced frictional effects contribute to the improved mechanical properties and toughness of the nanocomposites. The minimal frequency dependence of both G′ and G″ indicates that the nanocomposites exhibit stable rheological behavior across different frequencies, which is indicative of a robust and consistent network structure. The damping factor (tan δ) (Figure 10c) can also be used to evaluate the different viscoelastic behavior of the nanocomposites [60,61]. In the frequency range studied, the damping factor is less than 1 (Figure 10c and Table 4), which indicates that the elastic component (G′) of the pure polymer and nanocomposites was higher than the viscous modulus (G″), suggesting the gels showed solid-like behavior [62].

Figure 10d demonstrates that the complex viscosity of the nanocomposites exhibits pronounced shear-thinning behavior. The complex viscosity increases by a factor of 4 as the frequency decreases from 100 Hz to 0.01 Hz. For instance, at 0.1 Hz, the complex viscosity of the 1PLA is 1.3 × 10^4^ Pa·s, while it increases to 1.58 × 10^5^ Pa·s for the 7PLA (Table 4). This increase in complex viscosity with higher WO_3_ particle loading suggests enhanced network formation and consolidation within the PLA matrix. The shear-thinning behavior is characteristic of materials where the complex viscosity decreases with increasing shear rate, which is typical of gels and suspensions with a well-developed network structure. The presence of WO_3_ particles increases the material’s resistance to flow, contributing to a more structured and stable gel network. This effect is attributed to the interactions between the WO_3_ particles and polymer chains, which create a more complex internal network that affects the material’s flow behavior [63].

### 3.9. Antimicrobial Activity of PLA/WO_3_ Nanocomposites

The antimicrobial efficacy of PLA/WO_3_ nanocomposites was tested against Escherichia coli, *Staphylococcus aureus*, and *Candida albicans*. The assay mixed 5 mL of brain heart infusion broth (BHIB) with 0.1 g of each sample: PLA/WO_3_ nanocomposites, net PLA, and WO_3_ particles. PLA and WO_3_ served as controls to assess their individual antimicrobial effects. PLA alone showed no inhibition, confirming its lack of inherent antimicrobial properties, while the nanocomposites demonstrated enhanced activity [64,65,66]. This highlights the need for functional additives like WO_3_ particles to impart antimicrobial capabilities to PLA. However, the PLA/WO_3_ nanocomposites (1PLA, 3PLA, 5PLA, and 7PLA) did not exhibit inhibition zones against *E. coli* or *S. aureus*, suggesting resistance of these bacterial strains to the WO_3_ particles at the tested concentrations. This is consistent with Muzaffar et al. [67], who also observed limited antimicrobial activity of WO_3_/GO nanocomposites against these bacteria.

**Figure 11:**
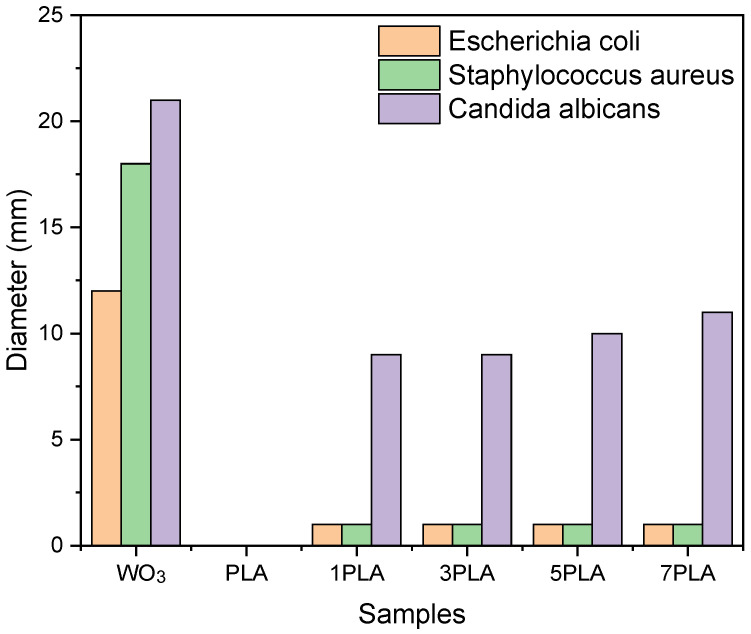
Antimicrobial activity of urchin-like WO_3_ particles, net PLA, and PLA/WO_3_ nanocomposites. PLA and WO_3_ served as controls to assess their individual antimicrobial effects.

In contrast, Candida albicans demonstrated notable sensitivity to the PLA/WO_3_ nanocomposites, with the 7PLA sample showing an inhibition zone of 11 mm, indicating effective antifungal activity (Figure 11). This enhanced activity against fungi compared to bacteria can be attributed to structural differences between fungal and bacterial cell walls. Fungi, such as C. albicans, have cell walls primarily composed of chitin and are less negatively charged than bacterial cell walls, which may hinder the penetration of antimicrobial agents. The WO_3_ particles likely exert their antifungal effects through the generation of reactive oxygen species that damage the less protective fungal cell wall. This differential susceptibility highlights the potential of the WO_3_ particles to be used in applications requiring antifungal properties, offering a valuable addition to the antimicrobial arsenal.

## 4. Conclusions

This study successfully developed and characterized PLA/WO_3_ nanocomposites with a distinct honeycomb-like morphology by incorporating urchin-like WO_3_ particles into the PLA matrix. FTIR analysis indicated that the interaction between the WO_3_ particles and PLA is physical rather than chemical. With increasing WO_3_ particle loading, more intense absorption peaks were observed. SEM micrographs revealed that the WO_3_ particles formed agglomerates within the honeycomb-like structure of the PLA matrix. The UV-Vis spectroscopy showed a significant increase in absorbance at 600 nm, suggesting improved UV blocking properties due to the presence of WO_3_. DSC analysis demonstrated a 20% increase in crystallinity with higher WO_3_ particle concentrations, although the glass transition and melting temperatures remained largely unaffected. The elimination of the melting shoulder at higher WO_3_ particle loading suggests a more homogeneous melting process. Thermal stability decreased proportionally with WO_3_ particle concentration, as evidenced by a reduced maximum degradation temperature. Rheological tests revealed that the incorporation of WO_3_ particles enhanced the mechanical properties of PLA, with the storage modulus increasing by 30 times at 7 wt% WO_3_, and improved gel stability. Antimicrobial tests demonstrated effective antifungal activity against *Candida albicans* but no significant antibacterial effects against *Escherichia coli* and *Staphylococcus aureus*. These findings highlight the promising potential of PLA/WO_3_ nanocomposites for advanced material applications, particularly where enhanced mechanical, thermal, and UV-blocking properties are desirable. It is important to note that dichloromethane (DCM) was used to dissolve PLA, facilitating a homogeneous solution and uniform nanoparticle dispersion. However, DCM’s toxicity poses risks for biomedical and food packaging applications. Therefore, complete removal of DCM through thorough processing is essential to ensure compliance with safety standards and prevent any residual solvent migration.

## Figures and Tables

**Table 1:** Composition and characteristics of the Neat PLA and PLA/WO_3_ nanocomposites. Mean pore diameter (µm) and mean pore area (µm). UV-blocking (%) was estimated using UV-Vis transmittance mode.

Samples	WO_3_ Conc.	Mean Pore Length (µm)	Mean Pore Area (µm)	UV-Blocking (%)
PLA	0 wt%	No pore	No pore	9 ± 1
1PLA	1 wt%	14.1 ± 1.0	4.17 ± 0.5	55 ± 2
3PLA	3 wt%	14.8 ± 1.2	5.59 ± 0.6	28 ± 3
5PLA	5 wt%	19.9 ± 1.5	7.40 ± 0.7	38 ± 2
7PLA	7 wt%	24.9 ± 1.8	9.22 ± 0.8	42 ± 2

**Table 2:** Thermal stability of neat PLA and PLA/WO_3_ nanocomposites, showing temperatures at 5%, 50%, and 95% weight loss, and the temperature of maximum weight loss rate.

Samples	WO_3_ Conc.	T_5%_ (°C)	T_50%_ (°C)	T_95%_ (°C)	T_max_ (°C)
PLA	0	269 ± 1.5	322 ± 1.2	339 ± 1.8	331.0 ± 1.0
1PLA	1	281 ± 1.3	318 ± 1.4	389 ± 1.7	330.2 ± 1.1
3PLA	3	281 ± 1.4	315 ± 1.3	331 ± 1.6	320.9 ± 1.2
5PLA	5	282 ± 1.2	310 ± 1.5	347 ± 1.8	317.4 ± 1.3
7PLA	7	268 ± 1.6	304 ± 1.7	326 ± 1.5	310.4 ± 1.2

**Table 3:** DSC results of the neat polymer and PLA/WO_3_ nanocomposites based on the second scan.

Samples	Tg (°C)	Tc (°C)	Tm_1_ (°C)	Tm_2_ (°C)	ΔHc (J/g)	ΔHm (J/g)	Crystallization Rate (%)
PLA	58.0 ± 1.2	115.0 ± 1.5	164.0 ± 1.3	169.0 ± 1.4	33.9 ± 1.3	37.1 ± 1.2	39.9 ± 1.5
1PLA	59.0 ± 0.8	112.0 ± 1.5	163.0 ± 1.0	168.0 ± 1.0	35.6 ± 1.7	36.2 ± 1.1	42.3 ± 1.0
3PLA	58.0 ± 1.1	110.0 ± 1.8	162.0 ± 1.2	168.0 ± 1.2	35.9 ± 1.5	38.7 ± 1.5	42.9 ± 1.8
5PLA	55.0 ± 1.4	104.0 ± 1.8	--	166.0 ± 1.5	28.5 ± 1.8	44.5 ± 1.7	50.5 ± 1.8

Abbreviations: glass transition temperature (T_g_), cold crystallization temperature (T_c_), melting temperature (T_m1_ and T_m2_), enthalpy of cold crystallization (ΔH_c_), melting enthalpy (ΔH_m_), crystallization rate (χ_c_).

**Table 4:** Rheological parameters of neat PLA and PLA/WO_3_ at 0.1 Hz.

Sample	G′ (Pa)	G″ (Pa)	tan(δ)	$\eta *\left(Pa\cdot s\right)$	Gel Temperature (°C)
PLA	3000 ± 150	956 ± 50	0.318 ± 0.015	4730 ± 220	74.3 ± 1.0
1PLA	8420 ± 250	2070 ± 75	0.246 ± 0.020	13,000 ± 460	68.9 ± 1.2
3PLA	14,500 ± 300	4730 ± 100	0.326 ± 0.025	21,800 ± 680	72.3 ± 1.3
5PLA	18,600 ± 400	3740 ± 125	0.201 ± 0.030	28,400 ± 740	76.0 ± 1.0
7PLA	101,000 ± 500	29,900 ± 150	0.296 ± 0.035	158,000 ± 790	78.5 ± 1.5

## Data Availability

The original contributions presented in the study are included in the article, further inquiries can be directed to the corresponding author/s.
